# Comparison of Hospitalization for Nonaffective Psychotic Disorders Among Refugee, Migrant, and Native-Born Adults in Sweden and Denmark

**DOI:** 10.1001/jamanetworkopen.2023.36848

**Published:** 2023-10-06

**Authors:** Alexis E. Cullen, Christopher J. de Montgomery, Marie Norredam, Jakob Bergström, Allan Krasnik, Heidi Taipale, Ellenor Mittendorfer-Rutz

**Affiliations:** 1 Department of Clinical Neuroscience, Division of Insurance Medicine, Karolinska Institutet, Stockholm, Sweden; 2 Department of Psychosis Studies, Institute of Psychiatry, Psychology & Neuroscience, King’s College London, London, United Kingdom; 3 Department of Public Health, Danish Research Centre for Migration, Ethnicity and Health, University of Copenhagen, Copenhagen, Denmark; 4 Section of Immigrant Medicine, Department of Infectious Diseases, University Hospital Hvidovre, Copenhagen, Denmark; 5 Niuvanniemi Hospital, Kuopio, Finland; 6 School of Pharmacy, University of Eastern Finland, Kuopio, Finland

## Abstract

**Importance:**

Determining whether migrants with nonaffective psychotic disorders (NAPDs) experience poorer outcomes after illness onset is essential to ensure adequate health care provision to these disadvantaged populations.

**Objective:**

To compare cumulative hospital days for NAPDs during the first 5 years of illness among refugee, nonrefugee, and second-generation migrants and their Swedish and Danish peers.

**Design, Setting, and Participants:**

This was a prospective cohort study of individuals treated for incident NAPDs in inpatient or outpatient settings between January 1, 2006, and December 31, 2013, and followed up for 5 years. This population-based study used Swedish and Danish national registries. Included participants were individuals in Sweden and Denmark, aged 18 to 35 years, treated for incident NAPDs. Data analyses were conducted from November 2022 to August 2023.

**Exposures:**

Population group (determined according to residency in either country, not both countries), categorized as refugee (migrants whose residence in Sweden or Denmark was registered as refugee status or family reunification with a refugee), nonrefugee (all other individuals born outside Sweden and Denmark), second generation (individuals born in Sweden or Denmark with at least 1 parent born abroad), or native born (individuals born in Sweden or Denmark with both parents born in these countries).

**Main Outcome and Measures:**

Total hospital days for NAPDs during the first 5 years of illness, analyzed using a hurdle model. Among those ever admitted, total number of admissions and mean admission length were examined.

**Results:**

In total, 7733 individuals in Sweden (mean [SD] age, 26.0 [5.1] years; 4919 male [63.6%]) and 8747 in Denmark (mean [SD] age 24.8 [5.0] years; 5324 male [60.9%]) were followed up for 5 years or until death or emigration. After adjusting for a range of sociodemographic and clinical factors, the odds of experiencing any hospital days for NAPD were significantly higher among migrant groups compared with their native-born peers (Sweden: second generation, odds ratio [OR], 1.17; 95% CI, 1.03-1.33; *P* = .01; nonrefugee migrant, OR, 1.45; 95% CI, 1.21-1.73; *P* < .001; refugee, OR, 1.25; 95% CI, 1.06-1.47; *P* = .009; Denmark: second generation, OR, 1.21; 95% CI, 1.05-1.40; *P* = .01; nonrefugee migrant, OR, 1.33; 95% CI, 1.14-1.55; *P* < .001). These odds were highest among nonrefugee (Sweden: OR, 2.53; 95% CI, 1.59-4.03; *P* < .001; Denmark: OR, 2.61; 95% CI, 1.70-4.01; *P* < .001) and refugee (Sweden: OR, 1.96; 95% CI, 1.43-2.69; *P* < .001; Denmark: OR, 2.14; 95% CI, 1.42-3.21; *P* < .001) migrants from Africa and those who had arrived within 3 to 5 years (Sweden: nonrefugee migrants, OR, 1.93; 95% CI, 1.26-2.95; *P* = .002; refugees, OR, 2.38; 95% CI, 1.46-3.88; *P* < .001; Denmark: nonrefugee migrants, OR, 1.66; 95% CI, 0.96-2.85; *P* = .07; refugees, OR, 3.40; 95% CI, 1.13-10.17; *P* = .03). Among those ever hospitalized, refugees in both countries (Sweden, incidence rate ratio [IRR], 1.30; 95% CI, 1.12-1.51; *P* < .001; Denmark, IRR, 1.47; 95% CI, 1.24-1.75; *P* < .001) and second-generation migrants in Denmark (IRR, 1.22; 95% CI, 1.07-1.39; *P* = .003) experienced more days hospitalized for NAPDs than native-born individuals.

**Conclusions and Relevance:**

In this prospective cohort study of individuals with NAPDs, results suggest that refugee, nonrefugee, and second-generation migrants experience more days hospitalized for these disorders than their native-born peers. Patterns were consistent across 2 countries with different models of psychosis care and immigration and integration policies.

## Key Points

**Question:**

Do refugee, nonrefugee, and second-generation migrants in Sweden and Denmark experience more days hospitalized for nonaffective psychotic disorders (NAPDs) during the first 5 years of illness than their native-born peers?

**Findings:**

In this cohort study including 7733 individuals in Sweden and 8747 in Denmark, all migrant groups were more likely than their Swedish and Danish peers to be hospitalized for NAPDs. Among those ever admitted, refugees and second-generation migrants experience more days hospitalized.

**Meaning:**

The excess risk of hospitalization for NAPDs among refugee, nonrefugee, and second-generation migrant groups is consistent across 2 countries with different models of early psychosis care and immigration and integration policies.

## Teaser

This cohort study investigates if second-generation, nonrefugee, and refugee migrants in Sweden and Denmark experience more days hospitalized for nonaffective psychotic disorders during the first 5 years of illness than their native-born peers.

## Introduction

Meta-analyses indicate that first- and second-generation migrants are at increased risk for developing schizophrenia and other nonaffective psychotic disorders (NAPDs)^1,2,3,4^ and that refugees are at even greater risk of these disorders than nonrefugee migrants.^5^ It is likely that both shared factors (eg, discrimination, language difficulties affecting health care access, socioeconomic disadvantage, and social isolation)^6^ and distinct factors (eg, exposure to war, conflict, famine, persecution, family fragmentation, and institutional accommodation on arrival, common among refugees)^7^ contribute to the excess risk of NAPD in these groups. Although these factors may plausibly influence illness course and lead to poorer outcomes among migrants, previous studies have yielded mixed findings for most outcomes (including functioning, remission, and engagement) with few examining refugees specifically.^8^ The paucity of studies examining refugees is particularly concerning given that their number worldwide has risen exponentially, increasing from 25.7 million to 32 million during the first half of 2022 alone.^9^ Studies examining long-term outcomes among refugees with NAPDs are essential to ensure adequate health care provision to these disadvantaged populations.

One important outcome is psychiatric hospitalization, which can be a distressing and stigmatizing experience^10^ and inevitably causes disruption to education, employment, and family life. Psychiatric hospitalization is driven by clinical need (ie, when individuals are deemed to be a danger to themselves or others), which may in turn be influenced by sociodemographic factors and delays or difficulties in accessing adequate health care in the early course of illness. Indeed, factors such as age, relationship status, ethnicity, and longer duration of untreated psychosis (DUP) have been associated with risk of hospitalization and longer length of stay among patients with psychosis.^11^ Given that refugees and nonrefugee migrants appear to underutilize specialist health care for several psychiatric disorders that are prevalent during the early stages of psychosis (eg, affective, anxiety, and substance use disorders),^12,13^ we might expect an excess risk of hospitalization for first-episode NAPD among migrants. Consistent with this hypothesis, 2 studies^14,15^ observed that refugee and first-generation, nonrefugee migrant groups were both more likely to be hospitalized during the first psychotic episode when compared with their native-born peers. Whether these disparities extend to subsequent admissions (when individuals are within the health care system) or length of stay (which might be prolonged due to clinical and/or sociodemographic factors) is less clear. Moreover, to our knowledge, no study has investigated whether second-generation migrants are equally disadvantaged.

To address these knowledge gaps, we compared patterns of hospitalization for NAPDs during the early course of illness among refugee, nonrefugee, and second-generation migrants and their native-born peers in Sweden and Denmark. Although there are some similarities in the general health care systems in these Scandinavian countries (both have decentralized health care systems with publicly financed psychiatric services^16^ and a similar number of psychiatric hospital beds per 100 000 inhabitants),^17^ these countries differ substantially in terms of the availability of early intervention in psychosis services. These specialist outpatient services have become widespread in Denmark over the past 2 decades^18^ but are scarce in Sweden despite recent recommendations for their introduction.^19^ Given that early intervention services aim to reduce DUP and improve long-term prognosis by providing prompt assessment and treatment for individuals with first-episode psychosis,^20^ health care disparities among migrants may be reduced in countries with early intervention (although if these groups have more difficulty accessing early intervention services, disparities may be further exacerbated). A further difference between these countries during the study period concerns immigration and integration policies, with Sweden historically implementing a more inclusionist approach (characterized by a higher immigrant population) than Denmark.^21^

In the present study, we used Swedish and Danish national registries to examine hospitalization for NAPDs during the first 5 years of illness; to reduce potential heterogeneity in illness course related to age of onset,^22,23,24^ study cohorts were restricted to those aged 18 to 35 years at first diagnosis. Our primary aim was to determine if refugee, nonrefugee, and second-generation migrants experienced more days hospitalized for NAPDs during the 5-year follow-up than their native-born peers and if patterns were similar in Sweden and Denmark. In subgroup analyses, we explored the impact of region of birth and duration of residence. Our secondary aim was to investigate whether any observed differences in hospital days were due to these migrant groups experiencing more frequent and/or longer admissions.

## Methods

### Data Sources

This study was approved by the regional ethical review board in Sweden. Data were obtained from multiple, nationwide registries in Sweden and Denmark (eTable 1 in Supplement 1). According to current Swedish and Danish regulations, the use of registry data for research purposes does not require informed consent from individuals held in these registries. Within each country, data were linked by the pseudonymized unique personal identification number assigned to all Swedish and Danish residents at birth or immigration. This study followed the Strengthening the Reporting of Observational Studies in Epidemiology (STROBE) reporting guidelines.

### Study Design and Population

The populations for this prospective cohort study were defined using the National Patient Registries in Sweden and Denmark according to the *International Statistical Classification of Diseases and Related Health Problems, Tenth Revision (ICD-10)*^25^ codes assigned at discharge or contact (for inpatient and outpatient treatments, respectively). We identified all individuals aged 18 to 35 years in Sweden and Denmark who received their first main diagnosis of NAPD (*ICD-10* codes F20-F29) in inpatient or specialist outpatient care between January 1, 2006, and December 31, 2013. To reduce the risk of differential misclassification across population groups, all individuals were required to have resided in the host country (Sweden or Denmark) for at least 3 full calendar years before cohort entry and have no recorded inpatient or outpatient contacts with a main diagnosis of NAPD during the previous 3 years (1080 days). We additionally excluded individuals who had any recorded purchases of antipsychotic medication (Anatomic Therapeutic Chemical classification codes N05A, omitting lithium N05AN) in the 15 months before cohort entry except for the final 3 months (which were permitted as we considered these to be part of the first treatment episode). Purchases during the 3 months before cohort entry were permitted as we considered these to be part of the first treatment episode. Individuals entered the cohort on the date of their first contact for NAPD (2006-2013, inclusive) and were followed up for 5 years or until death or emigration. Participant race and ethnicity data were not included in this study because such data are not available in the Swedish and Danish national registries.

### Determination of Refugee Status

Refugees included those whose grounds for residence in Sweden or Denmark as registered with immigration authorities was “refugee status” or “family reunification with a refugee.” In line with previous studies,^26,27^ as grounds of residence data were only available from 1993 in Denmark, we classified immigrants arriving from the major refugee-sending countries in the period 1986 to 1992 as refugees (eTable 1 in Supplement 1). All other individuals born abroad were classified as nonrefugee migrants; second-generation migrants were defined as individuals born in Sweden or Denmark with at least 1 parent who entered as a migrant. Those born in Sweden or Denmark with both parents born in these countries were classified as native born.

### Psychiatric Hospitalization During Follow-Up

Our primary outcome was the total number of days spent hospitalized during the 5-year follow-up with a main diagnosis of NAPD (*ICD-10* codes F20-F29) at discharge. Among those who had at least 1 hospital admission for NAPD during the 5-year follow-up, we also examined the total number of admissions and mean admission length (total number of hospital days/number of admissions).

### Measurement of Covariates

Analyses were adjusted for factors (or proxies for these factors) shown to influence hospitalization in first-episode psychosis populations.^11^ These included sociodemographic variables (age, gender, family situation, type of residence region, household income at age 18 years, level of education, and unemployment), measures of work disability indexing functional impairment associated with psychiatric and/or somatic conditions (sickness absence, disability pension), and clinical factors (prior treatment for mental disorders, somatic conditions, and suicide attempts; prior psychotropic medication; and NAPD diagnosis at first contact). Covariates were determined using the Swedish and Danish registries detailed in eTable 1 in Supplement 1. For nonrefugee migrant and refugee groups, we additionally measured region of birth and duration of residence.

### Statistical Analysis

Statistical analyses were performed from November 2022 to August 2023 in Sweden and Denmark separately using R software, versions 4.2.1 and 4.0.4 (R Core Team^28^), respectively. Population group differences in the primary outcome (total number of hospital days) were estimated using a hurdle model (glmmTMB package) suitable for analyzing count data with excess zeros.^29^ These 2-part models comprise a binary component and a truncated count component,^30^ allowing for the simultaneous modeling of the likelihood of having any hospital days (any vs 0) and the number of days hospitalized among those with at least 1 day. In the present study, we used a logistic (yielding odds ratios [ORs], inversed for interpretability) and truncated negative binomial model (yielding incidence rate ratios [IRRs]), respectively, for these 2 components. Both components included the same covariates with log (follow-up time) used as an offset in the model; observations with missing covariates were excluded from adjusted analyses. For the primary outcome, subgroup analyses were performed to explore the association of region of birth and duration of residence (analyses were not adjusted for covariates due to small group sizes). Analyses of secondary outcomes were restricted to those hospitalized at least once during the follow-up period. A truncated negative binomial model was used to compare population groups on the total number of admissions during the 5-year follow-up (offset = log [follow-up time]). Mean admission length was analyzed using a generalized linear model with gamma distribution and log-link function. All *P* values were 2-sided, and *P* < .05 was considered significant.

## Results

### Sample Characteristics at Cohort Entry

We identified 7733 individuals in Sweden (4222 native born [54.6%], 1648 second-generation migrants [21.3%], 861 nonrefugee migrants [11.1%], and 1002 refugees [13.0%]). Mean (SD) age for the total Swedish cohort was 26.0 (5.1) years; 4919 were male (63.6%) and 2814 were female (36.4%). We identified 8747 individuals in Denmark (6367 native born [72.8%], 935 second-generation migrants [10.7%], 904 nonrefugee migrants [10.3%], and 541 refugees [6.2%]). Mean (SD) age for the total Danish sample was 24.8 (5.0) years; 5324 were male (60.9%) and 3423 were female (39.1%). Participants from Sweden and Denmark received their first NAPD diagnosis between 2006 and 2013, inclusive, and were followed up for a mean (SD) of 4.9 (0.7) years and 4.9 (0.6) years, respectively (eTable 2 in Supplement 1 contains attrition rates). Sample characteristics are provided by country and population group in Table 1. There were some notable differences across population groups. In Sweden and Denmark, it was less common for both native-born and second-generation migrants to be married or cohabiting (Sweden: native born, 158 of 4222 [3.7%]; second generation, 75 of 1648 [4.6%]; Denmark: native born, 247 of 6367 [3.9%]; second generation, 40 of 935 [4.3%]) when compared with their nonrefugee and refugee counterparts (Sweden: nonrefugee, 180 of 861 (20.9%); refugee, 149 of 1002 (14.9%); Denmark: nonrefugee, 112 of 904 (12.4%); refugee, 50 of 541 (9.2%). Native-born individuals were also least likely to be residing in a city at cohort entry (Sweden: 1702 of 4222 [40.3%]; Denmark: 2519 of 6367 [39.6%]) and to have lived in a low-income household at age 18 years (Sweden: 476 of 4222 [11.1%]; Denmark: 1246 of 6367 [19.6%]). Treatment for other mental disorders in the 3 years before cohort entry and use of any psychotropic medication in the previous 6 months was most common among native-born individuals in both countries. With regard to country-level differences, schizophrenia (*ICD-10*: F20) was the most common diagnosis at cohort entry in Denmark (3129 of 8747 [35.8%]), whereas those with other psychotic disorders (*ICD-10*: F24-F29) formed the majority in Sweden (3622 of 7733 [46.8%]). Moreover, a higher proportion of individuals in Sweden than in Denmark received their first NAPD diagnosis in inpatient settings (4052 of 7733 [52.4%] vs 3061 of 8747 [35.0%], respectively). Data on region of birth and duration of residence are provided in eTable 3 in Supplement 1. Europe was the most common region of birth for Swedish nonrefugees (304 of 861 [35.3%]) and refugees (342 of 1002 [34.2%]) and Danish nonrefugees (374 of 904 [41.4%]), whereas most Danish refugees were born in West Asia (210 of 541 [38.8%]). Most refugees and nonrefugee migrants had resided in their host country for 11 or more years; those with a short duration of residency (3-5 years) were the minority across all groups, ranging from 3.7% (20 of 541 Danish refugees) to 15.6% (128 of 861 Swedish nonrefugee migrants).

**Table 1.** Sociodemographic and Clinical Characteristics of the Swedish and Danish Study Cohorts at Baseline

Demographic	No. (%)									
	Sweden^a^					Denmark^b^				
	Total (N = 7733)	Native born (n = 4222)	Second generation (n = 1648)	Nonrefugee migrant (n = 861)	Refugee (n = 1002)	Total (N = 8747)	Native born (n = 6367)	Second generation (n = 935)	Nonrefugee migrant (n = 904)	Refugee (n = 541)
Age, mean (SD), y^c^	26.0 (5.05)	25.9 (5.05)	25.6 (5.01)	28.1 (4.82)	25.6 (4.83)	24.8 (5.02)	24.5 (4.95)	24.4 (4.98)	26.7 (5.03)	26.2 (4.86)
Gender										
Man	4919 (63.6)	2676 (63.4)	1065 (64.6)	471 (54.7)	707 (70.6)	5324 (60.9)	3790 (59.5)	622 (66.5)	526 (58.2)	386 (71.3)
Woman	2814 (36.4)	1546 (36.6)	583 (35.4)	390 (45.3)	295 (29.4)	3423 (39.1)	2577 (40.5)	313 (33.5)	378 (41.8)	155 (28.7)
Family situation^d^										
Other	7171 (92.7)	4064 (96.3)	1573 (95.4)	681 (79.1)	853 (85.1)	8298 (94.9)	6120 (96.1)	895 (95.7)	792 (87.6)	491 (90.8)
Married or cohabiting	562 (7.3)	158 (3.7)	75 (4.6)	180 (20.9)	149 (14.9)	449 (5.1)	247 (3.9)	40 (4.3)	112 (12.4)	50 (9.2)
Type of residence region^d^										
City	3678 (47.6)	1702 (40.3)	892 (54.1)	491 (57.0)	593 (59.2)	3889 (44.5)	2519 (39.6)	550 (58.8)	531 (58.7)	289 (53.4)
Town/suburb	2879 (37.2)	1709 (40.5)	574 (34.8)	282 (32.8)	314 (31.3)	2605 (29.8)	2006 (31.5)	249 (26.6)	203 (22.5)	147 (27.2)
Rural	1176 (15.2)	811 (19.2)	182 (11.0)	88 (10.2)	95 (9.5)	2253 (25.8)	1842 (28.9)	136 (14.5)	170 (18.8)	105 (19.4)
Household income age 18 y										
≥60% of median	6243 (80.7)	3754 (88.9)	1349 (81.9)	512 (59.5)	628 (62.7)	6707 (76.7)	5121 (80.4)	634 (67.8)	671 (74.2)	281 (51.9)
<60% of median	1489 (19.3)	467 (11.1)	299 (18.1)	349 (40.5)	374 (37.3)	2040 (23.3)	1246 (19.6)	301 (32.2)	233 (25.8)	260 (48.1)
Education level^d^										
Compulsory	3903 (50.5)	1900 (45.0)	893 (54.2)	462 (53.7)	648 (64.7)	6255 (71.5)	4549 (71.4)	701 (75.0)	583 (64.6)	422 (78.0)
High school	2322 (30.0)	1415 (33.5)	484 (29.4)	188 (21.8)	235 (23.5)	2161 (24.7)	1589 (25.0)	205 (21.9)	261 (28.9)	106 (19.6)
University	1508 (19.5)	907 (21.5)	271 (16.4)	211 (24.5)	119 (11.9)	330 (3.8)	229 (3.6)	29 (3.1)	59 (6.5)	13 (2.4)
Unemployment days^d^										
None	5504 (71.2)	3142 (74.4)	1186 (72.0)	574 (66.7)	602 (60.1)	7157 (81.8)	5282 (83.0)	772 (82.6)	687 (76.0)	416 (76.9)
Any	2229 (28.8)	1080 (25.6)	462 (28.0)	287 (33.3)	400 (39.9)	1590 (18.2)	1085 (17.0)	163 (17.4)	217 (24.0)	125 (23.1)
Sickness absence days^d^										
≤30	7230 (93.5)	3891 (92.2)	1557 (94.5)	824 (95.7)	958 (95.6)	8102 (92.6)	5897 (92.6)	870 (93.0)	831 (91.9)	504 (93.2)
>30	503 (6.5)	331 (7.8)	91 (5.5)	37 (4.3)	44 (4.4)	645 (7.4)	470 (7.4)	65 (7.0)	73 (8.1)	37 (6.8)
Disability pension receipt^d^										
None	6788 (87.8)	3662 (86.7)	1434 (87.0)	776 (90.1)	916 (91.4)	8408 (96.1)	6105 (95.9)	906 (96.9)	880 (97.3)	517 (95.6)
Any	945 (12.2)	560 (13.3)	214 (13.0)	85 (9.9)	86 (8.6)	339 (3.9)	262 (4.1)	29 (3.1)	24 (2.7)	24 (4.4)
Prior mental disorder^e^										
None	3806 (49.2)	1953 (46.3)	803 (48.7)	499 (58.0)	551 (55.0)	5488 (62.7)	3897 (61.2)	619 (66.2)	600 (66.4)	372 (68.8)
Any	3927 (50.8)	2269 (53.7)	845 (51.3)	362 (42.0)	451 (45.0)	3259 (37.3)	2470 (38.8)	316 (33.8)	304 (33.6)	169 (31.2)
Prior somatic condition^e^										
None	3218 (41.6)	1769 (41.9)	689 (41.8)	362 (42.0)	398 (39.7)	3251 (37.2)	2400 (37.7)	338 (36.1)	328 (36.3)	185 (34.2)
Any	4515 (58.4)	2453 (58.1)	959 (58.2)	499 (58.0)	604 (60.3)	5496 (62.8)	3967 (62.3)	597 (63.9)	576 (63.7)	356 (65.8)
Prior suicide attempt^e^										
None	7224 (93.4)	3920 (92.8)	1532 (93.0)	815 (94.7)	957 (95.5)	8696 (99.4)	6327 (99.4)	>850 (NR)^f^	>850 (NR)	>450 (NR)
Any	509 (6.6)	302 (7.2)	116 (7.0)	46 (5.3)	45 (4.5)	51 (0.6)	40 (0.6)	<10 (NR)	<10 (NR)	<10 (NR)
Psychotropic medication^g^										
None	4948 (64.0)	2537 (60.1)	1059 (64.3)	606 (70.4)	746 (74.5)	5098 (58.3)	3541 (55.6)	614 (65.7)	589 (65.2)	354 (65.4)
Any	2785 (36.0)	1685 (39.9)	589 (35.7)	255 (29.6)	256 (25.5)	3649 (41.7)	2826 (44.4)	321 (34.3)	315 (34.8)	187 (34.6)
Diagnosis at first contact										
Schizophrenia	634 (8.2)	330 (7.8)	134 (8.1)	67 (7.8)	103 (10.3)	3129 (35.8)	2328 (36.6)	322 (34.4)	289 (32.0)	190 (35.1)
Schizotypal disorder	139 (1.8)	98 (2.3)	31 (1.9)	<10 (NR)	<10 (NR)	1680 (19.2)	1383 (21.7)	145 (15.5)	122 (13.5)	30 (5.5)
Delusional disorder	675 (8.7)	369 (8.7)	136 (8.3)	>70 (NR)	>70 (NR)	800 (9.1)	517 (8.1)	101 (10.8)	100 (11.1)	82 (15.2)
Acute or transient	2663 (34.4)	1561 (37.0)	489 (29.7)	294 (34.1)	319 (31.8)	2218 (25.4)	1465 (23.0)	277 (29.6)	301 (33.3)	175 (32.3)
Other psychotic disorder	3622 (46.8)	1864 (44.1)	858 (52.1)	410 (47.6)	490 (48.9)	920 (10.5)	674 (10.6)	90 (9.6)	92 (10.2)	64 (11.8)
First NAPD contact type										
Inpatient	4052 (52.4)	2132 (50.5)	863 (52.4)	503 (58.4)	554 (55.3)	3061 (35.0)	2133 (33.5)	339 (36.3)	390 (43.1)	199 (36.8)
Outpatient	3681 (47.6)	2090 (49.5)	785 (47.6)	358 (41.6)	448 (44.7)	5686 (65.0)	4234 (66.5)	596 (63.7)	514 (56.9)	342 (63.2)
Year of cohort entry										
2006	962 (12.4)	537 (12.7)	203 (12.3)	120 (13.9)	102 (10.2)	913 (10.4)	665 (10.4)	95 (10.2)	90 (10.0)	63 (11.6)
2007	880 (11.4)	468 (11.1)	181 (11.0)	108 (12.5)	123 (12.3)	947 (10.8)	672 (10.6)	86 (9.2)	111 (12.3)	78 (14.4)
2008	947 (12.2)	517 (12.2)	210 (12.7)	98 (11.4)	122 (12.2)	997 (11.4)	725 (11.4)	101 (10.8)	108 (11.9)	63 (11.6)
2009	957 (12.4)	549 (13.0)	187 (11.3)	92 (10.7)	129 (12.9)	1062 (12.1)	769 (12.1)	102 (10.9)	123 (13.6)	68 (12.6)
2010	950 (12.3)	516 (12.2)	194 (11.8)	107 (12.4)	133 (13.3)	1133 (13.0)	816 (12.8)	127 (13.6)	115 (12.7)	75 (13.9)
2011	969 (12.5)	561 (13.3)	195 (11.8)	106 (12.3)	107 (10.7)	1194 (13.7)	860 (13.5)	158 (16.9)	106 (11.7)	70 (12.9)
2012	1015 (13.1)	531 (12.6)	218 (13.2)	112 (13.0)	154 (15.4)	1217 (13.9)	894 (14.0)	133 (14.2)	127 (14.0)	63 (11.6)
2013	1053 (13.6)	543 (12.9)	260 (15.8)	118 (13.7)	132 (13.2)	1284 (14.7)	966 (15.2)	133 (14.2)	124 (13.7)	61 (11.3)

Abbreviations: NAPD, nonaffective psychotic disorder; NR, not reported.

^a^ Missing data Swedish cohort: household income at age 18 (n = 1).

^b^ Missing data Danish cohort: education level (n = 1).

^c^ Measured during year of cohort entry.

^d^ Measured on December 31 (Sweden) or September 30 (Denmark) in the calendar year before cohort entry.

^e^ Measured in the 3 relative years (1080 days) before cohort entry date.

^f^ NR to prevent determination of cells with counts less than 10 suppressed.

^g^ Measured in the 6 months (180 days) before cohort entry date.

### Total Hospital Days

Descriptive statistics for total hospital days attributable to NAPDs during the 5-year follow-up are provided in Table 2. For all groups, the proportion of individuals who spent at least 1 day in hospital was higher in Sweden (native born: 2741 of 4222 [64.9%]; second generation: 1130 of 1648 [68.6%]; nonrefugee: 635 of 861 [73.8%]; refugee: 714 of 1002 [71.3%]) than in Denmark (native born: 3443 of 6367 [54.1%]; second generation: 567 of 935 [60.6%]; nonrefugee: 570 of 904 [63.1%]; refugee: 316 of 541 [58.4%]). In contrast, among those ever hospitalized, the median (IQR) number of hospital days was substantially higher among all Danish groups (native born: 53.0 [15.0-139.0] days; second generation: 73.0 [19.0-180.5] days; nonrefugee: 62.0 [18.0-154.3] days; refugee: 83.5 [16.0-204.3] days) relative to their Swedish counterparts (native born: 31.0 [11.0-89.0] days; second generation: 38.0 [12.3-101.3] days; nonrefugee: 35.0 [12.0-91.0] days; refugee: 40.5 [13.0-115.8] days).

**Table 2.** Hospital Days for Nonaffective Psychotic Disorders During the First 5 Years of Illness Among Native-Born Individuals and Second-Generation, Nonrefugee, and Refugee Migrants in Sweden and Denmark^a^

Group	Any days in hospital (hurdle model—binary logistic component)					No. of days in hospital (hurdle model—truncated negative binomial component)					
	Descriptive, No. (%)	Crude analysis		Adjusted analysis		Descriptive		Crude analysis		Adjusted analysis	
		OR (95% CI)	*P* value	OR (95% CI)	*P* value	Mean	Median (IQR)	IRR (95% CI)	*P* value	IRR (95% CI)	*P* value
**Sweden**											
Population group											
Native born (n = 4222)	2741 (64.9)	1 [Reference]	NA	1 [Reference]	NA	99.6	31.0 (11.0-89.0)	1 [Reference]	NA	1 [Reference]	NA
Second generation (n = 1648)	1130 (68.6)	1.18 (1.04-1.33)	.008	1.17 (1.03-1.33)	.01	128.7	38.0 (12.3-101.3)	1.25 (1.10-1.41)	<.001	1.09 (0.96-1.22)	.18
Nonrefugee migrant (n = 861)	635 (73.8)	1.52 (1.29-1.79)	<.001	1.45 (1.21-1.73)	<.001	111.6	35.0 (12.0-91.0)	1.12 (0.97-1.31)	.13	1.09 (0.93-1.28)	.26
Refugee (n = 1002)	714 (71.3)	1.34 (1.15-1.56)	<.001	1.25 (1.06-1.47)	.009	154.6	40.5 (13.0-115.8)	1.55 (1.34-1.79)	<.001	1.30 (1.12-1.51)	<.001
**Denmark**											
Population group											
Native born (n = 6367)	3443 (54.1)	1 [Reference]	NA	1 [Reference]	NA	119.0	53.0 (15.0-139.0)	1 [Reference]	NA	1 [Reference]	NA
Second generation (n = 935)	567 (60.6)	1.31 (1.14-1.51)	<.001	1.21 (1.05-1.40)	.01	156.1	73.0 (19.0-180.5)	1.36 (1.19-1.55)	<.001	1.22 (1.07-1.39)	.003
Nonrefugee migrant (n = 904)	570 (63.1)	1.45 (1.26-1.67)	<.001	1.33 (1.14-1.55)	<.001	126.7	62.0 (18.0-154.3)	1.13 (0.99-1.29)	.06	1.07 (0.94-1.23)	.29
Refugee (n = 541)	316 (58.4)	1.19 (1.00-1.43)	.05	1.04 (0.86-1.26)	.65	174.4	83.5 (16.0-204.3)	1.52 (1.28-1.80)	<.001	1.47 (1.24-1.75)	<.001

Abbreviations: IRR, incidence rate ratio; NA, not applicable; OR, odds ratio.

^a^ Adjusted models include age, sex, family situation, region of residence, household income at age 18 years, education level, unemployment, sickness absence, disability pension, previous treatment for any mental disorder, previous treatment for somatic conditions, recent psychotropic medication purchases, psychotic disorder diagnosis at first contact, and calendar year at cohort entry.

Results for crude and adjusted hurdle models are provided in Table 2 (coefficients for all covariates provided in eTable 4 in Supplement 1). In the adjusted model, the odds of spending at least 1 day in hospital relative to their native-born peers were significantly higher among second-generation (OR, 1.17; 95% CI, 1.03-1.33; *P* = .01), nonrefugee (OR, 1.45; 95% CI, 1.21-1.73; *P* < .001), and refugee (OR, 1.25; 95% CI, 1.06-1.47; *P* = .009) migrant groups in Sweden and second-generation (OR, 1.21; 95% CI, 1.05-1.40; *P* = .01) and nonrefugee (OR, 1.33; 95% CI, 1.14-1.55; *P* < .001) migrants in Denmark. The number of days spent in hospital among those ever hospitalized was significantly higher among refugees in Sweden (IRR, 1.30; 95% CI, 1.12-1.51; *P* < .001) and second-generation and refugee groups in Denmark (IRR, 1.22; 95% CI, 1.07-1.39; *P *= .003 and IRR, 1.47; 95% CI, 1.24-1.75; *P* < .001 respectively) compared with native-born individuals in the adjusted models. In subgroup analyses (Figure; eTable 5 in Supplement 1), the odds of experiencing any hospital days (relative to native-born individuals) were more markedly increased in both countries among nonrefugee (Sweden: OR, 2.53; 95% CI, 1.59-4.03; *P* < .001; Denmark: OR, 2.61; 95% CI, 1.70-4.01; *P* < .001) and refugee (Sweden: OR, 1.96; 95% CI, 1.43-2.69; *P* < .001; Denmark: OR, 2.14; 95% CI, 1.42-3.21; *P* < .001) migrants who were born in Africa (Figure, A) and those who were within 3 to 5 years of arrival (Sweden: nonrefugee migrants, OR, 1.93; 95% CI, 1.26-2.95; *P* = .002; refugees, OR, 2.38; 95% CI, 1.46-3.88; *P* < .001; Denmark: nonrefugee migrants, OR, 1.66; 95% CI, 0.96-2.85; *P* = .07; refugees, OR, 3.40; 95% CI, 1.13-10.17; *P* = .03) (Figure, C); these patterns were not observed for number of days hospitalized (eTable 5 in Supplement 1).

**Figure.**
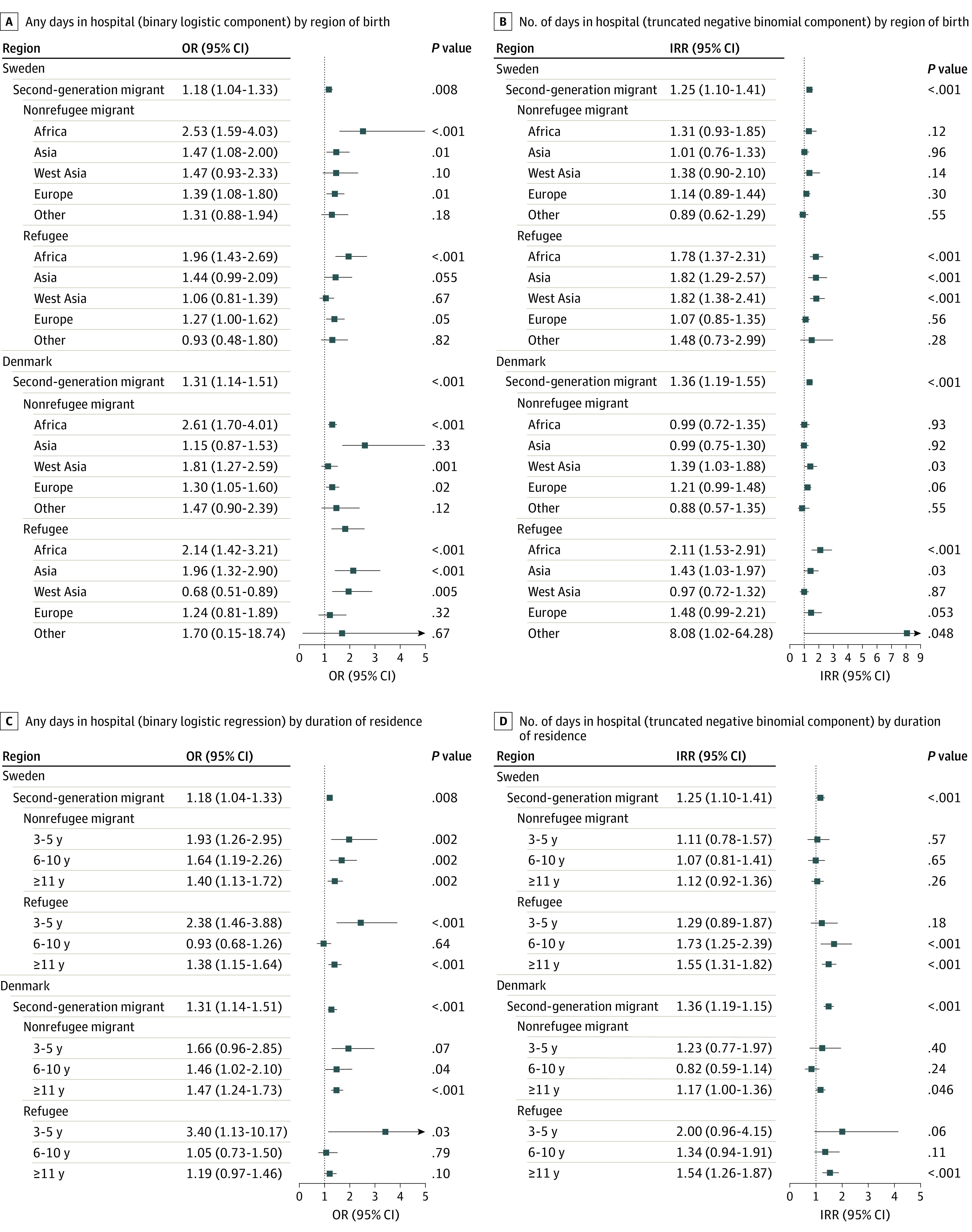
Hospital Days for Nonaffective Psychotic Disorders by Region of Birth and Duration of Residence Results of hurdle models applied to total hospital days for nonaffective psychotic disorders over the 5-year study period are shown by region of birth (any days [A] and, among those ever hospitalized, number of days [B]) and duration of residence (any days [C] and, among those ever hospitalized, number of days [D]) in Sweden and Denmark. These 2-component models estimate the odds of experiencing at least 1 day hospitalized (hurdle component − logistic model) and among those hospitalized, the number of days hospitalized (count component − truncated negative binomial model) for migrant groups relative to those who were native born (reference line).

### Frequency and Length of Hospitalization Among Those Admitted

Secondary outcomes were examined among those ever admitted for NAPD during the study period (Table 3; results for all covariates included in eTable 6 in Supplement 1). There were no significant group differences in the number of hospital admissions over the study period in either country (Danish refugees showed a significant increase relative to native-born individuals in the crude model only; IRR, 1.31; 95% CI, 1.03-1.66; *P* = .03). In contrast, in the adjusted models, mean days per admission were significantly increased among all migrant groups relative to native-born individuals (Sweden: second-generation, exponentiated beta [Exp(B)] = 1.12; 95% CI, 1.02-1.22; *P *= .02 ; refugee, Exp(B) = 1.34; 95% CI, 1.19-1.49; *P * < .001; Denmark: second-generation, Exp(B) = 1.27; 95% CI, 1.15-1.40; *P* < .001; nonrefugee, Exp(B) = 1.17; 95% CI, 1.06-1.29; *P *= .002; refugee, Exp(B) = 1.45; 95% CI, 1.27-1.65; *P* < .001) except for nonrefugee migrants in Sweden where a significant increase was observed in the crude model only (Exp[B] = 1.13; 95% CI, 1.00-1.27; *P *= .042).

**Table 3.** Number and Mean Duration of Hospitalizations for Nonaffective Psychotic Disorders During the First 5 Years of Illness Among Native-Born Individuals and Second-Generation, Nonrefugee, and Refugee Migrants in Sweden and Denmark

Group	No. of hospital admissions (truncated negative binomial model)^a^						Mean admission length (GLM with log-transformed response)^a^					
	Descriptive		Crude analysis		Adjusted analysis		Descriptive		Crude analysis		Adjusted analysis	
	Mean	Median (IQR)	IRR (95% CI)	*P* value	IRR (95% CI)	*P* value	Mean	Median (IQR)	Exp(B)^b^ (95% CI)	*P* value	Exp(B)^b^ (95% CI)	*P* value
**Sweden**												
Population group												
Native born	2.3	1.0 (1.0-3.0)	1 [Reference]	NA	1 [Reference]	NA	46.2	19.0 (8.0-38.5)	1 [Reference]	NA	1 [Reference]	NA
Second generation	2.4	2.0 (1.0-3.0)	1.14 (1.00-1.31)	.06	1.07 (0.93-1.22)	.34	62.5	21.0 (8.3-43.0)	1.23 (1.12-1.35)	<.001	1.12 (1.02-1.22)	.02
Nonrefugee migrant	2.2	1.0 (1.0-3.0)	1.01 (0.85-1.19)	.95	1.05 (0.88-1.25)	.61	52.5	21.0 (8.4-40.0)	1.13 (1.00-1.27)	.04	1.07 (0.94-1.20)	.30
Refugee	2.4	2.0 (1.0-3.0)	1.18 (1.00-1.39)	.05	1.06 (0.90-1.26)	.46	74.8	21.0 (9.0-49.3)	1.52 (1.36-1.69)	<.001	1.34 (1.19-1.49)	<.001
**Denmark**												
Population group												
Native born	3.6	2.0 (1.0-4.0)	1 [Reference]	NA	1 [Reference]	NA	38.7	22.5 (8.0-46.0)	1 [Reference]	NA	1 [Reference]	NA
Second generation	3.6	2.0 (1.0-4.0)	1.08 (0.90-1.29)	.41	1.07 (0.89-1.28)	.50	48.8	24.3 (9.8-57.1)	1.32 (1.19-1.45)	<.001	1.27 (1.15-1.40)	<.001
Nonrefugee migrant	3.5	2.0 (1.0-4.0)	1.05 (0.87-1.26)	.62	1.12 (0.93-1.35)	.25	39.6	22.2 (9.0-50.4)	1.16 (1.05-1.28)	.003	1.17 (1.06-1.29)	.002
Refugee	4.0	2.0 (1.0-4.0)	1.31 (1.03-1.66)	.03	1.27 (0.99-1.62)	.06	53.1	26.0 (9.4-58.1)	1.41 (1.24-1.60)	<.001	1.45 (1.27-1.65)	<.001

Abbreviations: GLM, generalized linear model; IRR, incidence rate ratio; NA, not applicable.

^a^ Adjusted models include age, sex, family situation, region of residence, household income at age 18, education level, unemployment, sickness absence, disability pension, previous treatment for any mental disorder, previous treatment for somatic conditions, recent psychotropic medication purchases, psychotic disorder diagnosis at first contact, and calendar year at cohort entry.

^b^ Indicates exponentiated unstandardized beta coefficient.

## Discussion

In this large population cohort study, we observed that all migrant groups (second generation, nonrefugee, and refugee) had increased levels of hospitalization for NAPDs compared with their native-born peers during the first 5 years of illness. Moreover, the odds of being hospitalized for NAPDs were most marked among nonrefugee migrants and refugees from Africa and those who were within 3 to 5 years of arrival. These patterns were consistent across Sweden and Denmark—2 countries that had markedly different models of early psychosis care and immigration and integration policies during the study period.

Our findings are consistent with those of previous studies that have observed an increased risk of hospitalization among nonrefugee migrants and refugees^14,15^ and with Swedish studies showing that the risk is even greater among those who had more recently migrated and individuals who had migrated from Africa.^12,14^ Importantly, we extended these findings by showing this was also true of second-generation migrants. Migrant status has been associated with delays in help seeking and a longer DUP,^31,32^ potentially due to a lack of awareness or understanding of local service provision, language barriers, and stigma.^33,34,35^ Structural factors, such as a lack of cultural competency within mental health care services,^36^ may also delay access to health care. Consistent with these findings and previous studies examining health care utilization,^12,13^ we observed that refugees and nonrefugee migrants were less likely than native-born individuals to have been treated for any other (nonpsychotic) psychiatric disorder or to have purchased psychotropic medication before cohort entry than their native-born peers. Such factors may plausibly lead to an increased need for hospitalization during early stages of psychotic illness among these groups. Although second-generation migrants may be less likely than these migrant groups to experience some of these factors (eg, language barriers and lack of knowledge of services), stigma and cultural differences may plausibly have contributed to the increased likelihood of hospitalization for NAPD in this group.

To our knowledge, this was the first study to examine cumulative hospital days for NAPD over an extended follow-up period in migrant groups. Among those who were admitted at least once, refugees in both countries and second-generation migrants in Denmark experienced more days hospitalized compared with their native-born peers. In our secondary analyses, we investigated whether this increase in cumulative hospital days was due to a higher number of admissions (potentially reflecting illness relapses that cannot be managed in outpatient care) or extended admissions (possibly attributable to greater illness severity, poor response to treatment, and/or difficulties organizing discharge) and found support for the latter. Indeed, mean admission length was significantly increased among all migrant groups when compared with their Swedish and Danish peers. Using registry data, we were not able to determine whether these extended stays were due to structural factors that could delay discharge (eg, a lack of secure and/or suitable accommodation or availability of an interpreter) or illness severity or treatment response. However, we adjusted for a range of factors that could be considered proxy measures of illness severity or burden at baseline with no changes to the overall pattern of results.

### Strengths and Limitations

The main strength of this study was that by using high-quality, national registry data from Sweden and Denmark, we were able to minimize loss to follow-up and adjust for a wide range of potential confounders. Nevertheless, some limitations must be noted. First, as noted previously, our use of registry data means that we were unable to capture clinical factors (eg, DUP and symptoms) that are likely to be strongly associated with risk and duration of hospital admission. As such, we lack knowledge of the mechanisms that underlie the differences that we observed. Second, by requiring that all individuals had to be residents in Sweden or Denmark for 3 years before cohort entry (necessary to reduce the potential for misclassification of incident cases among migrant groups), our study populations did not include refugees and migrants who became unwell soon after their arrival. Third, as grounds of residence information was only available in Denmark from 1993, migrants arriving between 1986 to 1992 were categorized as refugee or nonrefugee depending on their country of origin (with those arriving from major refugee-sending countries classified as refugees). However, similar imputation methods have been used in previous studies^26^ and by Statistics Denmark.^37^ Finally, our findings may lack generalizability to countries without publicly financed health care systems.

## Conclusions

In 2 countries which were characterized by markedly different models of early psychosis care and immigration and integration policies during the study period, in this cohort study, we observed that hospitalization for NAPDs during the first 5 years of illness was elevated in refugee, nonrefugee, and second-generation migrants compared with their native-born peers. Our findings, which extend those observed in previous studies, suggest that further research is needed to disentangle the complex processes that contribute to increased risk of hospitalization and prolonged stay within these disadvantaged populations. Importantly, different factors may be at play in these 3 migrant groups.

## Notes

Supplement 1. **eTable 1.** Variable Definitions and Data Sources **eTable 2.** Follow-up in the Swedish and Danish Study Cohorts During the 5-Year Study Period **eTable 3.** Region of Birth and Duration of Residence for Nonrefugee Migrant and Refugee Populations **eTable 4.** Days Spent in Hospital for Nonaffective Psychotic Disorders During the First 5 Years of Illness Among Native-Born Individuals and Second-Generation, Nonrefugee, and Refugee Migrants in Sweden and Denmark: Results of Fully Adjusted Hurdle Models With Coefficients Provided for All Covariates **eTable 5.** Days Spent in Psychiatric Hospital for Nonaffective Psychotic Disorders During the First 5 Years of Illness Among Native-Born Individuals and Second-Generation, Nonrefugee, and Refugee Migrants in Sweden and Denmark by Region of Birth and Duration of Residence **eTable 6.** Number and Mean Duration of Hospitalizations for Nonaffective Psychotic Disorders During the First 5 Years of Illness (for Those Admitted at Least Once) Among Native-Born Individuals and Second-Generation, Nonrefugee, and Refugee Migrants in Sweden and Denmark: Results of Fully Adjusted Models With Coefficients Provided for All Covariates
eReferences

Supplement 2.
Data Sharing Statement

## References

[1] Jongsma HE, Turner C, Kirkbride JB, Jones PB. International incidence of psychotic disorders, 2002-17: a systematic review and meta-analysis. Lancet Public Health. 2019;4(5):e229-e244. doi:10.1016/S2468-2667(19)30056-8 31054641 PMC6693560

[2] Selten JP, van der Ven E, Termorshuizen F. Migration and psychosis: a meta-analysis of incidence studies. Psychol Med. 2020;50(2):303-313. doi:10.1017/S0033291719000035 30722795 PMC7083571

[3] Cantor-Graae E, Selten JP. Schizophrenia and migration: a meta-analysis and review. Am J Psychiatry. 2005;162(1):12-24. doi:10.1176/appi.ajp.162.1.12 15625195

[4] Bourque F, van der Ven E, Malla A. A meta-analysis of the risk for psychotic disorders among first- and second-generation immigrants. Psychol Med. 2011;41(5):897-910. doi:10.1017/S0033291710001406 20663257

[5] Brandt L, Henssler J, Müller M, Wall S, Gabel D, Heinz A. Risk of psychosis among refugees: a systematic review and meta-analysis. JAMA Psychiatry. 2019;76(11):1133-1140. doi:10.1001/jamapsychiatry.2019.1937 31411649 PMC6694397

[6] Morgan C, Hutchinson G. The social determinants of psychosis in migrant and ethnic minority populations: a public health tragedy. Psychol Med. 2010;40(5):705-709. doi:10.1017/S003329170800490X 19335938

[7] Porter M, Haslam N. Predisplacement and postdisplacement factors associated with mental health of refugees and internally displaced persons: a meta-analysis. JAMA. 2005;294(5):602-612. doi:10.1001/jama.294.5.602 16077055

[8] Maguire J, Sizer H, Mifsud N, O’Donoghue B. Outcomes for migrants with a first episode of psychosis: a systematic review. Schizophr Res. 2020;222:42-48. doi:10.1016/j.schres.2020.05.048 32561236

[9] The United Nations Refugee Agency. Midyear trends. Accessed September 10, 2023. https://www.unhcr.org/mid-year-trends

[10] Fenton K, Larkin M, Boden ZVR, Thompson J, Hickman G, Newton E. The experiential impact of hospitalization in early psychosis: service-user accounts of inpatient environments. Health Place. 2014;30:234-241. doi:10.1016/j.healthplace.2014.09.013 25460906

[11] Ajnakina O, Stubbs B, Francis E, . Hospitalization and length of hospital stay following first-episode psychosis: systematic review and meta-analysis of longitudinal studies. Psychol Med. 2020;50(6):991-1001. doi:10.1017/S0033291719000904 31057129

[12] Björkenstam E, Helgesson M, Norredam M, Sijbrandij M, de Montgomery CJ, Mittendorfer-Rutz E. Differences in psychiatric care utilization between refugees, nonrefugee migrants and Swedish-born youth. Psychol Med. 2022;52(7):1365-1375. doi:10.1017/S0033291720003190 32914741

[13] Barghadouch A, Kristiansen M, Jervelund SS, Hjern A, Montgomery E, Norredam M. Refugee children have fewer contacts to psychiatric healthcare services: an analysis of a subset of refugee children compared to Danish-born peers. Soc Psychiatry Psychiatr Epidemiol. 2016;51(8):1125-1136. doi:10.1007/s00127-016-1260-1 27333980

[14] Katsampa D, Akther SF, Hollander AC, Dal H, Dalman C, Kirkbride JB. Inequalities in psychiatric service use and mortality by migrant status following a first diagnosis of psychotic disorder: a Swedish cohort study of 1.3m people. Schizophr Bull Open. 2021;2(1):sgab009. doi:10.1093/schizbullopen/sgab009 33898991 PMC8052494

[15] Rodrigues R, Beswick A, Anderson KK. Psychiatric hospitalization following psychosis onset: a retrospective cohort study using health administrative data. Early Interv Psychiatry. 2020;14(2):235-240. doi:10.1111/eip.12893 31696672

[16] NOMESCO Nordic. Financing of health care in the Nordic countries. Accessed September 10, 2023. https://norden.diva-portal.org/smash/get/diva2:968753/FULLTEXT01.pdf

[17] Eurostat. Mental health care—psychiatric hospital beds. Accessed February 26, 2023. https://ec.europa.eu/eurostat/web/products-eurostat-news/-/edn-20201009-1

[18] Nordentoft M, Melau M, Iversen T, . From research to practice: how OPUS treatment was accepted and implemented throughout Denmark. Early Interv Psychiatry. 2015;9(2):156-162. doi:10.1111/eip.12108 24304658

[19] von Malortie S, Cronqvist E, Ringbäck G, . [New national guidelines for the treatment of schizophrenia in Sweden]. Lakartidningen. 2019;116:116. 30694520

[20] McGorry PD, Killackey E, Yung A. Early intervention in psychosis: concepts, evidence, and future directions. World Psychiatry. 2008;7(3):148-156. doi:10.1002/j.2051-5545.2008.tb00182.x 18836582 PMC2559918

[21] de Montgomery CJ, Norredam M, Krasnik A, . Labor market marginalization in young refugees and their majority peers in Denmark and Sweden: the role of common mental disorders and secondary school completion. PLoS One. 2022;17(2):e0263450. doi:10.1371/journal.pone.0263450 35171929 PMC8849515

[22] Selvendra A, Baetens D, Trauer T, Petrakis M, Castle D. First episode psychosis in an adult area mental health service-a closer look at early and late-onset first episode psychosis. Australas Psychiatry. 2014;22(3):235-241. doi:10.1177/1039856214532558 24811714

[23] Fernandes NA, Martins MA, Gasparinho RF, . Late-onset first-episode psychosis: does it have a better outcome? Prim Care Companion CNS Disord. 2022;24(4):21br03073. doi:10.4088/PCC.21br03073 35926860

[24] Ballageer T, Malla A, Manchanda R, Takhar J, Haricharan R. Is adolescent-onset first-episode psychosis different from adult onset? J Am Acad Child Adolesc Psychiatry. 2005;44(8):782-789. doi:10.1097/01.chi.0000164591.55942.ea 16034280

[25] World Health Organization. ICD-10: International Statistical Classification of Diseases and Related Health Problems. World Health Organization; 2004.

[26] Damm AP. Ethnic enclaves and immigrant labor market outcomes: quasi-experimental evidence. J Labor Econ. 2009;27(2):281-314. doi:10.1086/599336

[27] de Montgomery CJ, Petersen JH, Jervelund SS. Diminishing social inequality between refugee children and their peers growing up in Denmark. J Ethn Migr Stud. 2018;46:1301-1329. doi:10.1080/1369183X.2018.1526061

[28] R Core Team. A Language and Environment for Statistical Computing: R Foundation for Statistical Computing, Vienna, Austria. Accessed September 6, 2023. https://www.r-project.org/

[29] Feng CX. A comparison of zero-inflated and hurdle models for modeling zero-inflated count data. J Stat Distrib Appl. 2021;8(1):8. doi:10.1186/s40488-021-00121-4 34760432 PMC8570364

[30] Min Y, Agresti A. Random-effects models for repeated measures of zero-inflated count data. Stat Model. 2005;5(1):1-19. doi:10.1191/1471082X05st084oa

[31] Nerhus M, Berg AO, Haram M, Kvitland LR, Andreassen OA, Melle I. Migrant background and ethnic minority status as predictors for duration of untreated psychosis. Early Interv Psychiatry. 2015;9(1):61-65. doi:10.1111/eip.12106 24225002

[32] Boonstra N, Sterk B, Wunderink L, Sytema S, De Haan L, Wiersma D. Association of treatment delay, migration and urbanicity in psychosis. Eur Psychiatry. 2012;27(7):500-505. doi:10.1016/j.eurpsy.2011.05.001 21705200

[33] Lindert J, Schouler-Ocak M, Heinz A, Priebe S. Mental health, health care utilization of migrants in Europe. Eur Psychiatry. 2008;23(suppl 1):14-20. doi:10.1016/S0924-9338(08)70057-9 18371575

[34] Jensen NK, Norredam M, Priebe S, Krasnik A. How do general practitioners experience providing care to refugees with mental health problems—a qualitative study from Denmark. BMC Fam Pract. 2013;14:17. doi:10.1186/1471-2296-14-17 23356401 PMC3568406

[35] Satinsky E, Fuhr DC, Woodward A, Sondorp E, Roberts B. Mental health care utilization and access among refugees and asylum seekers in Europe: a systematic review. Health Policy. 2019;123(9):851-863. doi:10.1016/j.healthpol.2019.02.007 30850148

[36] Bhui K. A Refugee Rose of competencies and capabilities for mental healthcare of refugees. BJPsych Open. 2022;8(2):e45. doi:10.1192/bjo.2022.11 35152939 PMC8867853

[37] Danmarks Statistik. Opholdsgrundlag. Accessed August 17, 2023. https://www.dst.dk/da/TilSalg/Forskningsservice/Dokumentation/hoejkvalitetsvariable/opholdsgrundlag

