# Supplementary material for: Comparison of Hospitalization for Nonaffective Psychotic Disorders Among Refugee, Migrant, and Native-Born Adults in Sweden and Denmark
Source: JAMA Netw Open. 2023 Oct 6;6(10):e2336848. doi: 10.1001/jamanetworkopen.2023.36848 (PMC10559176; doi:10.1001/jamanetworkopen.2023.36848)
Supplement: Supplement 1. — eTable 1. Variable Definitions and Data Sources eTable 2. Follow-up in the Swedish and Danish Study Cohorts During the 5-Year Study Period eTable 3. Region of Birth and Duration of Residence for Nonrefugee Migrant and Refugee Populations eTable 4. Days Spent in Hospital for Nonaffective Psychotic Disorders During the First 5 Years of Illness Among Native-Born Individuals and Second-Generation, Nonrefugee, and Refugee Migrants in Sweden and Denmark: Results of Fully Adjusted Hurdle Models With Coefficients Provided for All Covariates eTable 5. Days Spent in Psychiatric Hospital for Nonaffective Psychotic Disorders During the First 5 Years of Illness Among Native-Born Individuals and Second-Generation, Nonrefugee, and Refugee Migrants in Sweden and Denmark by Region of Birth and Duration of Residence eTable 6. Number and Mean Duration of Hospitalizations for Nonaffective Psychotic Disorders During the First 5 Years of Illness (for Those Admitted at Least Once) Among Native-Born Individuals and Second-Generation, Nonrefugee, and Refugee Migrants in Sweden and Denmark: Results of Fully Adjusted Models With Coefficients Provided for All Covariates eReferences [file jamanetwopen-e2336848-s001.pdf]

Supplementary Online Content

Cullen AE, de Montgomery CJ, Norredam M, et al. Comparison of hospitalization for nonaffective psychotic disorders among refugee, migrant, and native-born adults in Sweden and Denmark. *JAMA Netw Open*. 2023;6(9):e2336848.
doi:10.1001/jamanetworkopen.2023.36848

eTable 1. Variable Definitions and Data Sources
eTable 2. Follow-up in the Swedish and Danish Study Cohorts During the 5-Year Study Period
eTable 3. Region of Birth and Duration of Residence for Nonrefugee Migrant and Refugee Populations
eTable 4. Days Spent in Hospital for Nonaffective Psychotic Disorders During the First 5 Years of Illness Among Native-Born Individuals and Second-Generation, Nonrefugee, and Refugee Migrants in Sweden and Denmark: Results of Fully Adjusted Hurdle Models with Coefficients Provided for All Covariates
eTable 5. Days Spent in Psychiatric Hospital for Nonaffective Psychotic Disorders During the First 5 Years of Illness Among Native-Born Individuals and Second-Generation, Nonrefugee, and Refugee Migrants in Sweden and Denmark by Region of Birth and Duration of Residence
eTable 6. Number and Mean Duration of Hospitalizations for Nonaffective Psychotic Disorders During the First 5 Years of Illness (for Those Admitted at Least Once) Among Native-Born Individuals and Second-Generation, Nonrefugee, and Refugee Migrants in Sweden and Denmark: Results of Fully Adjusted Models with Coefficients Provided for all Covariates
eReferences

This supplementary material has been provided by the authors to give readers additional information about their work.

© 2023 Cullen AE et al. *JAMA Network Open*.

eTable 1. Variable Definitions and Data Sources

Variable	Definition	Swedish register	Danish Register
**Sociodemographic**			
Age	Years of age during the calendar year of cohort entry	LISA	BEF
Gender	Measured on the 31st of December in the year prior to cohort entry, categorised as men vs. women	LISA	BEF
Family situation	Measured on the 31st of December in the year prior to cohort entry, categorised as married/cohabiting vs. other	LISA	BEF
Type of residence region	Measured on the 31st of December in the year prior to cohort entry, categorised as cities vs. towns/suburbs vs. rural, according to EUROSTAT's degree of urbanization [DEGURBA] classification of local administrative units	LISA	BEF
Household income	Measured at age 18 years (equivalised using the square root method to account for household size [1]) indicating level of material deprivation during adolescence, categorised as annual income >= 60th percentile of the median that year vs. annual income < 60th percentile of the median	LISA	IND
Level of education	Measured on the 30th September (Denmark) / 31st of December (Sweden) in the year prior to cohort entry, categorised as compulsory vs. high school vs. college/university	LISA	BUE
Unemployment	Number of unemployment days during the calendar year prior to cohort entry, categorised as none vs. any	LISA	IDAP
Sickness absence	Gross sickness absence days during the calendar year prior to cohort entry, categorised as ≤30 vs. >30 days	MiDAS	SGDP
Disability pension	Receipt of disability pension during the calendar year prior to cohort entry, categorised as none vs. any	MiDAS	IND, SOCP
**Clinical**			
Treatment for psychiatric disorder	Any inpatient/specialist outpatient treatment with a main diagnosis of any non-psychotic psychiatric disorder (ICD-10 codes F00-F99, excluding F20-29) during the three relative years (1080 days) prior to cohort entry, categorised as no vs. yes	NPR	NPR
Treatment for somatic conditions	Any inpatient/specialist outpatient treatment with a main diagnosis of any somatic condition (ICD-10 codes A-Z, excluding F, O80, X60-X84, Y10-Y34, and Z) during the three relative years (1080 days) prior to cohort entry, categorised as no vs. yes	NPR	NPR
Treatment for suicide attempts	Any inpatient/specialist outpatient treatment for suicide attempt or event of undetermined intent (ICD-10 codes X60-X84, Y10-Y34) during the three relative years (1080 days) prior to cohort entry, categorised as no vs. yes	NPR	NPR
Psychotropic medication	Dispensations of any psychotropic medications, including antipsychotics, lithium, anxiolytics, benzodiazepines, hypnotics, Z drugs, antidepressants, and mood stabilisers (ACT codes N05A, N05B, N05C, N06A, N03AF01, N03AG01, N03AX09, and N05AN01) measured during the six months (180 days) prior to cohort entry, categorised as no vs. yes	PDR	DPR
NAPD diagnosis	NAPD diagnosis at first contact (assigned at discharge/contact for inpatient/outpatient treatments, respectively), categorised as schizophrenia (ICD-10: F20) vs. schizotypal disorder (ICD-10: F21) vs. delusional disorder (ICD-10: F22) vs. acute or transient psychotic disorder (ICD-10: F23) vs. other (ICD-10: F24-F29)	NPR	NPR
**Other**			
Refugee status [a]	Grounds for residence in Sweden/Denmark registered with immigration authorities as "refugee status" or "family reunification with a refugee", categorised as no vs. yes	STATIV	IEPE, OPHG

© 2023 Cullen AE et al. *JAMA Network Open*.

Variable	Definition	Swedish register	Danish Register
Non-refugee status [a]	Born in country other than host country, categorised as no vs. yes	LISA	BEF, IEPE, OPHG
Second-generation migrant status	Born in Sweden/Denmark with one or more parents born in country other than host country, categorised as no vs. yes	LISA	BEF, IEPE, OPHG
Death	Death during five-year study period, categorised as no vs. yes	CDR	DODSAASG
Emigration	Emigration during five-year study period, categorised as no vs. yes	LISA	BEF
Region-of-birth [b]	Defined according to the United Nations Standard Country or Area Codes for Statistical Use [2] as Africa vs. Western Asia vs. Other Asia vs. Europe	LISA	IEPE
Duration of residence [b]	Duration of residence in host country, categorised as 3-5 years vs. 6-10 years vs. 11+ years	LISA	VNDS

[a] In Denmark, grounds of residence data were only available from 1993, therefore migrants arriving before 1993 were categorised as non-refugee migrant or refugee based on their originating country. In line with previous publications,[3] which used data from Statistics Denmark to identify the largest refugee-sending countries between 1986 and 1993, we classified individuals arriving from Afghanistan, Lebanon, Iran, Iraq, Somalia, Sri Lanka and Vietnam as refugees based on the rationale that these countries accounted for the vast majority of refugees within this period. In the current study, this approximation method was used to classify individuals who migrated to Denmark between 1986-1992, accounting for 28.10% of individuals classified as non-refugee migrants and 24.21% of those classified as refugees in the Danish cohort.

[b] Determined for refugees and non-refugee migrant groups.

Abbreviations: ACT: Anatomic Therapeutic Chemical; BEF: Population Registry; BUE: Population's Education Register; CDR: Cause of Death Register; DODSAASG: Register of Causes of Death; DPR: Drug Prescription Registry; ICD-10: International Classification of Diseases – version 10; IDAP: Database for Labour Market Research; IEPE: Immigrants and descendants; IND: Income Registry; LISA: Longitudinal Integration Database for Health Insurance and Labor Market Studies; MiDAS: Micro-Data for Analyses of Social Insurance; NAPD: non-affective psychotic disorder; NPR: National Patient Register; OPHG: Grounds of Residence (supplementary dataset from the Immigration Agency covering 1993-1996); PDR: Prescribed Drug Register; SGDP: Sickness Absence Register; SOCP: Social Pensions; STATIV: Longitudinal Database for Integration Studies; VNDS: Historical walks.

© 2023 Cullen AE et al. *JAMA Network Open*.

eTable 2. Follow-up in the Swedish and Danish Study Cohorts During the 5-Year Study Period

	Sweden					Denmark				
	Total (n = 7733)	Native born (n = 4222)	Second generation (n = 1648)	Nonrefugee migrant (n = 861)	Refugee (n = 1002)	Total (n = 8747)	Native born (n = 6367)	Second generation (n = 935)	Nonrefugee migrant (n = 904)	Refugee (n = 541)
**Mean (SD) follow-up time, years**	4.85 (0.71)	4.87 (0.67)	4.85 (0.69)	4.75 (0.90)	4.84 (0.74)	4.90 (0.58)	4.92 (0.49)	4.85 (0.70)	4.81 (0.81)	4.79 (0.76)
**Follow-up status at 5 years, n (%)**										
Followed-up	7,303 (94.4)	4,028 (95.4)	1,555 (94.4)	776 (90.1)	944 (94.2)	8,366 (95.6)	6,151 (96.6)	881 (94.2)	841 (93.0)	493 (91.1)
Lost due to death	262 (3.4)	150 (3.6)	63 (3.8)	20 (2.3)	29 (2.9)	143 (1.6)	105 (1.6)	16 (1.7)	12 (1.3)	10 (1.8)
Lost due to emigration	168 (2.2)	44 (1.0)	30 (1.8)	65 (7.5)	29 (2.9)	357 (4.1)	199 (3.1)	51 (5.5)	62 (6.9)	45 (8.3)
Lost due to death or emigration	430 (5.6)	194 (4.6)	93 (5.6)	85 (9.9)	58 (5.8)	381 (4.4)	216 (3.4)	54 (5.8)	63 (7.0)	48 (8.9)

Abbreviations: SD: standard deviation.

© 2023 Cullen AE et al. *JAMA Network Open*.

eTable 3. Region of Birth and Duration of Residence for Nonrefugee Migrant and Refugee Populations

	Sweden				Denmark			
	Nonrefugee migrant (n = 861)		Refugee (n = 1,002)		Nonrefugee migrant (n = 904)		Refugee (n = 541)	
**Region of birth**								
Africa	125	(14.5)	236	(23.6)	114	(12.6)	116	(21.4)
Asia	216	(25.1)	143	(14.3)	198	(21.9)	119	(22.0)
Europe	304	(35.3)	342	(34.2)	374	(41.4)	91	(16.8)
Other	123	(14.3)	38	(3.8)	71	(7.9)	3	(0.6)
West Asia	93	(10.8)	243	(24.3)	141	(15.6)	210	(38.8)
**Duration of residence**								
3-5 years	128	(15.6)	108	(10.8)	59	(7.0)	20	(3.7)
6-10 years	210	(25.7)	185	(18.5)	128	(15.3)	123	(22.7)
11+ years	480	(58.7)	709	(70.8)	652	(77.7)	398	(73.6)

All data are shown as n (%).

Missing data: Country of birth, Danish nonrefugee migrants (n = 6); Danish refugees (n = 2). Duration of residence, Swedish non-refugee migrants (n = 43); Danish non-refugee migrants (n = 65).

© 2023 Cullen AE et al. *JAMA Network Open*.

eTable 4. Days Spent in Hospital for Nonaffective Psychotic Disorders During the First 5 Years of Illness Among Native-Born Individuals and Second-Generation, Nonrefugee, and Refugee Migrants in Sweden and Denmark: Results of Fully Adjusted Hurdle Models with Coefficients Provided for All Covariates

	Any days in hospital (binary logistic component)				Number of days in hospital (truncated negative binomial component)			
	Sweden		Denmark		Sweden		Denmark	
	OR (95% CI)	*P* value	OR (95% CI)	*P* value	IRR (95%CI)	*P* value	IRR (95%CI)	*P* value
**Population group**								
Native born	1.00 [Ref]		1.00 [Ref]		1.00 [Ref]		1.00 [Ref]	
Second generation	**1.17 (1.03–1.33)**	**0.014**	**1.21 (1.05–1.40)**	**0.011**	1.09 (0.96–1.22)	0.176	**1.22 (1.07–1.39)**	**0.003**
Nonrefugee migrant	**1.45 (1.21–1.73)**	**<0.001**	**1.33 (1.14–1.55)**	**<0.001**	1.09 (0.93–1.28)	0.263	1.07 (0.94–1.23)	0.285
Refugee	**1.25 (1.06–1.47)**	**0.009**	1.04 (0.86–1.26)	0.653	**1.30 (1.12–1.51)**	**<0.001**	**1.47 (1.24–1.75)**	**<0.001**
**Age (years)**	1.01 (1.00–1.02)	0.216	**0.99 (0.98–1.00)**	**0.007**	**0.98 (0.97–0.99)**	**<0.001**	0.99 (0.98–1.00)	0.164
**Gender**								
Men	1.00 [Ref]		1.00 [Ref]		1.00 [Ref]		1.00 [Ref]	
Women	1.00 (0.90–1.11)	0.963	**0.89 (0.81–0.98)**	**0.015**	**0.77 (0.69–0.85)**	**<0.001**	**0.84 (0.77–0.91)**	**<0.001**
**Family situation**								
Other	1.00 [Ref]		1.00 [Ref]		1.00 [Ref]		1.00 [Ref]	
Married/cohabiting	**0.66 (0.54–0.81)**	**<0.001**	0.93 (0.76–1.16)	0.530	0.87 (0.72–1.06)	0.166	**0.68 (0.56–0.83)**	**<0.001**
**Type of residence region**								
City	1.00 [Ref]		1.00 [Ref]		1.00 [Ref]		1.00 [Ref]	
Town/suburb	0.97 (0.87–1.08)	0.566	**0.79 (0.71–0.88)**	**<0.001**	0.96 (0.86–1.06)	0.386	**0.82 (0.74–0.90)**	**<0.001**
Rural	0.91 (0.78–1.05)	0.196	**0.78 (0.70–0.87)**	**<0.001**	**0.82 (0.71–0.94)**	**0.004**	**0.76 (0.68–0.84)**	**<0.001**
**Household income age 18**								
>=60 pct of median	1.00 [Ref]		1.00 [Ref]		1.00 [Ref]		1.00 [Ref]	
< 60 pct of median	**1.15 (1.01–1.32)**	**0.037**	1.06 (0.95–1.18)	0.319	1.12 (0.99–1.26)	0.069	1.04 (0.94–1.15)	0.464
**Education level**								
Compulsory	1.00 [Ref]		1.00 [Ref]		1.00 [Ref]		1.00 [Ref]	
High school	**1.16 (1.03–1.30)**	**0.016**	**1.24 (1.11–1.39)**	**<0.001**	0.97 (0.87–1.09)	0.641	0.98 (0.89–1.09)	0.769
University	**1.27 (1.09–1.48)**	**0.002**	**1.87 (1.43–2.44)**	**<0.001**	0.88 (0.77–1.01)	0.070	1.04 (0.84–1.28)	0.719

© 2023 Cullen AE et al. *JAMA Network Open*.

	Any days in hospital (binary logistic component)				Number of days in hospital (truncated negative binomial component)			
	Sweden		Denmark		Sweden		Denmark	
	OR (95% CI)	*P* value	OR (95% CI)	*P* value	IRR (95%CI)	*P* value	IRR (95%CI)	*P* value
**Unemployment days**								
None	1.00 [Ref]		1.00 [Ref]		1.00 [Ref]		1.00 [Ref]	
Any	**1.19 (1.06–1.34)**	**0.003**	0.99 (0.88–1.12)	0.928	**1.11 (1.01–1.23)**	**0.037**	0.94 (0.85–1.05)	0.308
**Sickness absence days**								
<= 30	1.00 [Ref]		1.00 [Ref]		1.00 [Ref]		1.00 [Ref]	
>30	**0.81 (0.66–0.98)**	**0.035**	**0.72 (0.61–0.86)**	**<0.001**	0.99 (0.81–1.22)	0.936	1.03 (0.87–1.23)	0.699
**Disability pension receipt**								
None	1.00 [Ref]		1.00 [Ref]		1.00 [Ref]		1.00 [Ref]	
Any	**0.82 (0.70–0.96)**	**0.012**	0.95 (0.75–1.20)	0.662	**1.36 (1.15–1.60)**	**<0.001**	**1.39 (1.10–1.74)**	**0.005**
**Prior mental disorder**								
None	1.00 [Ref]		1.00 [Ref]		1.00 [Ref]		1.00 [Ref]	
Any	0.99 (0.89–1.11)	0.902	**1.25 (1.14–1.37)**	**<0.001**	**1.22 (1.10–1.34)**	**<0.001**	**1.25 (1.14–1.36)**	**<0.001**
**Prior somatic condition**								
None	1.00 [Ref]		1.00 [Ref]		1.00 [Ref]		1.00 [Ref]	
Any	0.91 (0.82–1.01)	0.075	**1.17 (1.07–1.28)**	**<0.001**	0.99 (0.90–1.09)	0.849	0.96 (0.88–1.05)	0.347
**Prior suicide attempt**								
None	1.00 [Ref]		1.00 [Ref]		1.00 [Ref]		1.00 [Ref]	
Any	**1.52 (1.23–1.87)**	**<0.001**	**2.34 (1.20–4.56)**	**0.013**	1.11 (0.92–1.34)	0.271	1.43 (0.90–2.25)	0.127
**Psychotropic medication**								
None	1.00 [Ref]		1.00 [Ref]		1.00 [Ref]		1.00 [Ref]	
Any	**0.56 (0.50–0.62)**	**<0.001**	**1.51 (1.38–1.66)**	**<0.001**	**0.67 (0.60–0.74)**	**<0.001**	**0.88 (0.81–0.96)**	**0.004**
**Diagnosis at first contact**								
Schizophrenia	1.00 [Ref]		1.00 [Ref]		1.00 [Ref]		1.00 [Ref]	
Schizotypal disorder	**0.26 (0.17–0.38)**	**<0.001**	**0.49 (0.43–0.55)**	**<0.001**	**0.34 (0.29–0.41)**	**<0.001**	**0.62 (0.54–0.71)**	**<0.001**
Delusional disorder	**0.57 (0.45–0.72)**	**<0.001**	0.98 (0.84–1.15)	0.837	**0.65 (0.51–0.83)**	**<0.001**	**0.74 (0.64–0.86)**	**<0.001**
Acute or transient	**1.57 (1.30–1.91)**	**<0.001**	**1.79 (1.58–2.01)**	**<0.001**	**0.46 (0.27–0.78)**	**0.004**	**0.63 (0.57–0.69)**	**<0.001**
Other	1.09 (0.91–1.31)	0.361	**0.68 (0.59–0.79)**	**<0.001**	**0.52 (0.44–0.62)**	**<0.001**	**0.62 (0.54–0.73)**	**<0.001**
**Year of cohort entry**								

© 2023 Cullen AE et al. *JAMA Network Open*.

	Any days in hospital (binary logistic component)				Number of days in hospital (truncated negative binomial component)			
	**Sweden**		**Denmark**		**Sweden**		**Denmark**	
	**OR (95% CI)**	***P* value**	**OR (95% CI)**	***P* value**	**IRR (95%CI)**	***P* value**	**IRR (95%CI)**	***P* value**
2006	1.00 [Ref]		1.00 [Ref]		1.00 [Ref]		1.00 [Ref]	
2007	0.86 (0.70–1.05)	0.139	1.00 (0.83–1.21)	0.977	0.94 (0.78–1.14)	0.543	1.06 (0.89–1.26)	0.486
2008	0.85 (0.70–1.04)	0.112	0.91 (0.76–1.10)	0.350	0.88 (0.73–1.06)	0.179	0.92 (0.77–1.09)	0.341
2009	0.97 (0.79–1.19)	0.774	1.02 (0.85–1.23)	0.852	0.84 (0.70–1.00)	0.055	**0.82 (0.69–0.97)**	**0.021**
2010	**0.82 (0.67–0.99)**	**0.044**	1.11 (0.93–1.34)	0.257	0.87 (0.73–1.05)	0.146	**0.84 (0.71–0.99)**	**0.043**
2011	0.94 (0.77–1.15)	0.548	1.00 (0.83–1.20)	0.977	0.90 (0.75–1.08)	0.275	**0.78 (0.66–0.92)**	**0.004**
2012	0.94 (0.77–1.14)	0.508	0.90 (0.75–1.07)	0.235	**0.82 (0.69–0.98)**	**0.032**	**0.76 (0.65–0.90)**	**0.001**
2013	0.99 (0.81–1.20)	0.889	**0.81 (0.68–0.97)**	**0.021**	0.98 (0.82–1.17)	0.839	**0.74 (0.62–0.87)**	**<0.001**

Abbreviations: OR: odds ratio; CI: confidence interval; IRR: incidence rate ratio

© 2023 Cullen AE et al. *JAMA Network Open*.

eTable 5. Days Spent in Psychiatric Hospital for Nonaffective Psychotic Disorders During the First 5 Years of Illness Among Native-Born Individuals and Second-Generation, Nonrefugee, and Refugee Migrants in Sweden and Denmark by Region of Birth and Duration of Residence

	**Any days in hospital (binary logistic component)**				**Number of days in hospital (truncated negative binomial component)**			
	**Sweden**		**Denmark**		**Sweden**		**Denmark**	
	**OR (95% CI)**	***P* value**	**OR (95% CI)**	***P* value**	**IRR (95%CI)**	***P* value**	**IRR (95%CI)**	***P* value**
**Region of birth**								
Native born	1.00 [Ref]		1.00 [Ref]		1.00 [Ref]		1.00 [Ref]	
Second generation	**1.18 (1.04–1.33)**	**0.008**	**1.31 (1.14–1.51)**	**<0.001**	**1.25 (1.10–1.41)**	**<0.001**	**1.36 (1.19–1.55)**	**<0.001**
Nonrefugee migrant Africa	**2.53 (1.59–4.03)**	**<0.001**	**2.61 (1.70–4.01)**	**<0.001**	1.31 (0.93–1.85)	0.123	0.99 (0.72–1.35)	0.931
Nonrefugee migrant Asia	**1.47 (1.08–2.00)**	**0.014**	1.15 (0.87–1.53)	0.331	1.01 (0.76–1.33)	0.959	0.99 (0.75–1.30)	0.924
Nonrefugee migrant West Asia	1.47 (0.93–2.33)	0.103	**1.81 (1.27–2.59)**	**0.001**	1.38 (0.90–2.10)	0.139	**1.39 (1.03–1.88)**	**0.031**
Nonrefugee migrant Europe	**1.39 (1.08–1.80)**	**0.012**	1.30 (1.05–1.60)	0.017	1.14 (0.89–1.44)	0.300	1.21 (0.99–1.48)	0.060
Nonrefugee migrant Other	1.31 (0.88–1.94)	0.184	1.47 (0.90–2.39)	0.120	0.89 (0.62–1.29)	0.545	0.88 (0.57–1.35)	0.553
Refugee Africa	**1.96 (1.43–2.69)**	**<0.001**	**2.14 (1.42–3.21)**	**<0.001**	**1.78 (1.37–2.31)**	**<0.001**	**2.11 (1.53–2.91)**	**<0.001**
Refugee Asia	1.44 (0.99–2.09)	0.055	**1.96 (1.32–2.90)**	**<0.001**	**1.82 (1.29–2.57)**	**<0.001**	**1.43 (1.03–1.97)**	**0.030**
Refugee West Asia	1.06 (0.81–1.39)	0.672	**0.68 (0.51–0.89)**	**0.005**	**1.82 (1.38–2.41)**	**<0.001**	0.97 (0.72–1.32)	0.866
Refugee Europe	1.27 (1.00–1.62)	0.050	1.24 (0.81–1.89)	0.318	1.07 (0.85–1.35)	0.560	1.48 (0.99–2.21)	0.053
Refugee Other	0.93 (0.48–1.80)	0.821	1.70 (0.15–18.74)	0.666	1.48 (0.73–2.99)	0.277	**8.08 (1.02–64.28)**	**0.048**
**Duration of residence**								
Native born	1.00 [Ref]		1.00 [Ref]		1.00 [Ref]		1.00 [Ref]	
Second generation	**1.18 (1.04–1.33)**	**0.008**	**1.31 (1.14–1.51)**	**<0.001**	**1.25 (1.10–1.41)**	**<0.001**	**1.36 (1.19–1.55)**	**<0.001**
Nonrefugee migrant 3-5 years	**1.93 (1.26–2.95)**	**0.002**	1.66 (0.96–2.85)	0.068	1.11 (0.78–1.57)	0.567	1.23 (0.77–1.97)	0.396
Nonrefugee migrant 6-10 years	**1.64 (1.19–2.26)**	**0.002**	**1.46 (1.02–2.10)**	**0.040**	1.07 (0.81–1.41)	0.646	0.82 (0.59–1.14)	0.240
Nonrefugee migrant 11+ years	**1.40 (1.13–1.72)**	**0.002**	**1.47 (1.24–1.73)**	**<0.001**	1.12 (0.92–1.36)	0.257	**1.17 (1.00–1.36)**	**0.046**
Refugee 3-5 years	**2.38 (1.46–3.88)**	**<0.001**	**3.40 (1.13–10.17)**	**0.029**	1.29 (0.89–1.87)	0.175	2.00 (0.96–4.15)	0.063
Refugee 6-10 years	0.93 (0.68–1.26)	0.640	1.05 (0.73–1.50)	0.790	**1.73 (1.25–2.39)**	**<0.001**	1.34 (0.94–1.91)	0.105
Refugee 11+ years	**1.38 (1.15–1.64)**	**<0.001**	1.19 (0.97–1.46)	0.102	**1.55 (1.31–1.82)**	**<0.001**	**1.54 (1.26–1.87)**	**<0.001**

Abbreviations: OR: odds ratio; CI: confidence interval; IRR: incidence rate ratio

All models are crude (no covariates).

© 2023 Cullen AE et al. *JAMA Network Open*.

eTable 6. Number and Mean Duration of Hospitalizations for Nonaffective Psychotic Disorders During the First 5 Years of Illness (for Those Admitted at Least Once) Among Native-Born Individuals and Second-Generation, Nonrefugee, and Refugee Migrants in Sweden and Denmark: Results of Fully Adjusted Models with Coefficients Provided for all Covariates

	Number of hospital admissions (truncated negative binomial model)				Mean admission length (GLM with log-transformed response)			
	**Sweden**		**Denmark**		**Sweden**		**Denmark**	
	**IRR (95%CI)**	***P* value**	**IRR (95%CI)**	***P* value**	**B (95% CI)**	***P* value**	**B (95% CI)**	***P* value**
**Population group**								
Native born	1.00 [Ref]		1.00 [Ref]		1.00 [Ref]		1.00 [Ref]	
Second generation	1.07 (0.93–1.22)	0.340	1.07 (0.89–1.28)	0.497	**1.12 (1.02–1.22)**	**0.016**	**1.27 (1.15–1.40)**	**<0.001**
Nonrefugee migrant	1.05 (0.88–1.25)	0.614	1.12 (0.93–1.35)	0.246	1.07 (0.94–1.20)	0.304	**1.17 (1.06–1.29)**	**0.002**
Refugee	1.06 (0.90–1.26)	0.463	1.27 (0.99–1.62)	0.056	**1.34 (1.19–1.49)**	**<0.001**	**1.45 (1.27–1.65)**	**<0.001**
**Age (years)**	**0.97 (0.96–0.98)**	**<0.001**	**0.97 (0.96–0.99)**	**<0.001**	1.00 (0.99–1.01)	0.622	**1.01 (1.00–1.02)**	**0.001**
**Gender**								
Men	1.00 [Ref]		1.00 [Ref]		1.00 [Ref]		1.00 [Ref]	
Women	1.01 (0.90–1.13)	0.880	0.97 (0.86–1.10)	0.610	**0.77 (0.71–0.83)**	**<0.001**	**0.82 (0.77–0.88)**	**<0.001**
**Family situation**								
Other	1.00 [Ref]		1.00 [Ref]		1.00 [Ref]		1.00 [Ref]	
Married/cohabiting	0.80 (0.64–1.01)	0.056	**0.73 (0.56–0.95)**	**0.021**	0.92 (0.80–1.07)	0.291	0.87 (0.75–1.01)	0.066
**Type of residence region**								
City	1.00 [Ref]		1.00 [Ref]		1.00 [Ref]		1.00 [Ref]	
Town/suburb	0.89 (0.79–1.00)	0.052	1.01 (0.88–1.16)	0.852	1.00 (0.93–1.08)	0.964	**0.83 (0.77–0.89)**	**<0.001**
Rural	0.91 (0.78–1.07)	0.251	**0.86 (0.74–1.00)**	**0.046**	**0.83 (0.74–0.92)**	**<0.001**	**0.85 (0.79–0.92)**	**<0.001**
**Household income age 18**								
>=60 pct of median	1.00 [Ref]		1.00 [Ref]		1.00 [Ref]		1.00 [Ref]	
< 60 pct of median	1.13 (0.98–1.30)	0.081	1.09 (0.94–1.25)	0.249	**1.12 (1.02–1.23)**	**0.015**	1.04 (0.96–1.12)	0.343
**Education level**								
Compulsory	1.00 [Ref]		1.00 [Ref]		1.00 [Ref]		1.00 [Ref]	
High school	0.97 (0.85–1.09)	0.586	0.89 (0.77–1.03)	0.110	1.06 (0.97–1.15)	0.183	1.08 (0.99–1.16)	0.071
University	**0.81 (0.70–0.95)**	**0.009**	0.94 (0.70–1.26)	0.674	0.96 (0.86–1.06)	0.385	1.03 (0.88–1.21)	0.701
**Unemployment days**								

© 2023 Cullen AE et al. *JAMA Network Open*.

	Number of hospital admissions (truncated negative binomial model)				Mean admission length (GLM with log-transformed response)			
	Sweden		Denmark		Sweden		Denmark	
	IRR (95%CI)	*P* value	IRR (95%CI)	*P* value	B (95% CI)	*P* value	B (95% CI)	*P* value
None	1.00 [Ref]		1.00 [Ref]		1.00 [Ref]		1.00 [Ref]	
Any	**1.26 (1.12–1.41)**	**<0.001**	0.95 (0.82–1.11)	0.546	1.03 (0.95–1.11)	0.494	0.99 (0.91–1.08)	0.837
**Sickness absence days**								
<= 30	1.00 [Ref]		1.00 [Ref]		1.00 [Ref]		1.00 [Ref]	
>30	1.18 (0.93–1.49)	0.174	0.94 (0.74–1.19)	0.609	1.12 (0.96–1.31)	0.156	**1.15 (1.00–1.31)**	**0.043**
**Disability pension receipt**								
None	1.00 [Ref]		1.00 [Ref]		1.00 [Ref]		1.00 [Ref]	
Any	1.14 (0.95–1.37)	0.163	**1.44 (1.04–1.99)**	**0.027**	**1.49 (1.32–1.69)**	**<0.001**	**1.27 (1.07–1.50)**	**0.006**
**Prior mental disorder**								
None	1.00 [Ref]		1.00 [Ref]		1.00 [Ref]		1.00 [Ref]	
Any	**1.22 (1.09–1.37)**	**<0.001**	**1.30 (1.15–1.47)**	**<0.001**	**1.31 (1.22–1.41)**	**<0.001**	1.06 (1.00–1.14)	0.067
**Prior somatic condition**								
None	1.00 [Ref]		1.00 [Ref]		1.00 [Ref]		1.00 [Ref]	
Any	1.09 (0.98–1.22)	0.119	**1.21 (1.07–1.36)**	**0.003**	**0.89 (0.83–0.96)**	**0.002**	**0.87 (0.81–0.93)**	**<0.001**
**Prior suicide attempt**								
None	1.00 [Ref]		1.00 [Ref]		1.00 [Ref]		1.00 [Ref]	
Any	1.09 (0.88–1.35)	0.436	**2.14 (1.09–4.20)**	**0.026**	1.12 (0.97–1.30)	0.123	0.98 (0.70–1.39)	0.925
**Psychotropic medication**								
None	1.00 [Ref]		1.00 [Ref]		1.00 [Ref]		1.00 [Ref]	
Any	0.90 (0.80–1.02)	0.102	1.05 (0.93–1.18)	0.449	**0.63 (0.58–0.68)**	**<0.001**	**0.83 (0.78–0.89)**	**<0.001**
**Diagnosis at first contact**								
Schizophrenia	1.00 [Ref]		1.00 [Ref]		1.00 [Ref]		1.00 [Ref]	
Schizotypal disorder	**0.46 (0.25–0.87)**	**0.017**	**0.56 (0.47–0.68)**	**<0.001**	**0.48 (0.32–0.72)**	**<0.001**	**0.80 (0.73–0.88)**	**<0.001**
Delusional disorder	0.90 (0.68–1.18)	0.439	0.83 (0.67–1.02)	0.077	**0.65 (0.54–0.79)**	**<0.001**	**0.72 (0.64–0.81)**	**<0.001**
Acute or transient	**0.73 (0.60–0.90)**	**0.003**	**0.81 (0.70–0.93)**	**0.003**	**0.30 (0.26–0.34)**	**<0.001**	**0.62 (0.57–0.67)**	**<0.001**
Other	0.85 (0.70–1.04)	0.124	**0.70 (0.56–0.86)**	**<0.001**	**0.45 (0.39–0.52)**	**<0.001**	**0.65 (0.58–0.73)**	**<0.001**
**Year of cohort entry**								
2006	1.00 [Ref]		1.00 [Ref]		1.00 [Ref]		1.00 [Ref]	

© 2023 Cullen AE et al. *JAMA Network Open*.

	Number of hospital admissions (truncated negative binomial model)				Mean admission length (GLM with log-transformed response)			
	Sweden		Denmark		Sweden		Denmark	
	IRR (95%CI)	*P* value	IRR (95%CI)	*P* value	B (95% CI)	*P* value	B (95% CI)	*P* value
2007	0.96 (0.78–1.19)	0.737	1.20 (0.94–1.54)	0.135	0.90 (0.78–1.03)	0.128	0.88 (0.78–1.01)	0.064
2008	0.92 (0.75–1.14)	0.467	1.09 (0.86–1.40)	0.471	0.87 (0.76–1.01)	0.060	**0.80 (0.70–0.91)**	**<0.001**
2009	0.97 (0.79–1.20)	0.806	1.11 (0.87–1.40)	0.406	**0.83 (0.73–0.96)**	**0.010**	**0.76 (0.67–0.87)**	**<0.001**
2010	0.99 (0.80–1.22)	0.901	1.05 (0.83–1.33)	0.676	0.87 (0.76–1.00)	0.058	**0.78 (0.69–0.89)**	**<0.001**
2011	0.87 (0.71–1.08)	0.206	1.12 (0.89–1.42)	0.329	0.90 (0.78–1.03)	0.127	**0.70 (0.62–0.80)**	**<0.001**
2012	0.96 (0.78–1.18)	0.667	1.23 (0.97–1.56)	0.084	**0.75 (0.66–0.87)**	**<0.001**	**0.67 (0.59–0.76)**	**<0.001**
2013	1.09 (0.89–1.34)	0.397	1.21 (0.96–1.54)	0.108	0.90 (0.79–1.03)	0.133	**0.57 (0.50–0.65)**	**<0.001**

Abbreviations: IRR: incidence rate ratio; CI: confidence interval; GLM: Generalized linear model; *β*: beta coefficient.

© 2023 Cullen AE et al. *JAMA Network Open*.

eReferences

1. OCED. Compare your income – Methodology and conceptual issues. https://www.oecd.org/statistics/Compare-your-income-methodology.pdf. Published 2020. Accessed 31st January 2023.
2. United Nations Statistics Division. Standard Country or Area Codes for Statistical Use (M49). In. New York.
3. Damm AP. Ethnic Enclaves and Immigrant Labor Market Outcomes: Quasi-Experimental Evidence. *Journal of Labor Economics.* 2009;27(2):281-314.

© 2023 Cullen AE et al. *JAMA Network Open*.
