# Supplementary material for: Comparison of Hospitalization for Nonaffective Psychotic Disorders Among Refugee, Migrant, and Native-Born Adults in Sweden and Denmark
Source: JAMA Netw Open. 2023 Oct 6;6(10):e2336848. doi: 10.1001/jamanetworkopen.2023.36848 (PMC10559176; doi:10.1001/jamanetworkopen.2023.36848)
Supplement: Supplement 2. — Data Sharing Statement [file jamanetwopen-e2336848-s002.pdf]

Data Sharing Statement

Cullen. Comparison of Hospitalization for Nonaffective Psychotic Disorders Among Refugee, Migrant, and Native-Born Adults in Sweden and Denmark. *JAMA Netw Open*. Published October 03, 2023. doi:10.1001/jamanetworkopen.2023.36848

Data
Data available: No

Additional Information
Explanation for why data not available: These data cannot be made publicly available due to privacy regulations. According to the General Data Protection Regulation, the Swedish law SFS 2018:218, the Swedish Data Protection Act, the Swedish Ethical Review Act, and the Public Access to Information and Secrecy Act, these types of sensitive data can only be made available for specific purposes, including research, that meets the criteria for access to this type of sensitive and confidential data as determined by a legal review.
